# Gap between Willingness and Behaviors: Understanding the Consistency of Farmers’ Green Production in Hainan, China

**DOI:** 10.3390/ijerph191811351

**Published:** 2022-09-09

**Authors:** Dan Qiao, Shuting Xu, Tao Xu, Qinchuan Hao, Zhen Zhong

**Affiliations:** 1 Management School, Hainan University, Haikou 570228, China; 2 School of Agricultural Economics and Rural Development, Renmin University of China, Beijing 100872, China

**Keywords:** agricultural green development, farmer production, behavior–willingness deviation, influencing factors

## Abstract

The green transformation of production modes plays an essential role in the sustainable development of China’s agriculture and the modernization process, but there is often a deviation between farmers’ behavior and their willingness regarding green production. This paper analyzed the factors influencing the deviation of farmers’ green production behaviors from their willingness, along with their hierarchical logic structure, using the ordered logit model and ISM model with field survey data of 436 households in Hainan Province. The results show that: (1) there are deviations between farmers’ green production behavior and willingness; (2) age, number of dependents, peer influence, and social networks aggravate farmers’ green production behavior–willingness deviation, while ethnicity, education, land fragmentation, agricultural expenditure, land transfer, neighborhood learning, and green production cognition mitigate the deviation; (3) among the significant influencing factors, farmers’ perceptions of green production, peer influence, land transfer, and agricultural expenditure are the direct surface factors, while neighborhood learning, land fragmentation, and number of dependents are the middle indirect factors, and farmers’ education, social networks, age, and ethnicity are the deep-rooted factors. This study sheds more light and detail on the understanding of the factors influencing farmers’ green production behavior–willingness deviation, and provides more practical and relevant guidance for the agricultural green development in tropical China.

## 1. Introduction

The deterioration of the ecological environment has become a global crisis threatening the survival and development of human beings. Examples include the degradation of arable land, the pollution of air and water systems, the excessive use of chemical fertilizers, deforestation, and animals facing extinction, which directly or indirectly impact human wellbeing. China also faces these problems during rapid economic development and industrialization [1,2]. Since the reform and opening up, the rapid economic and social development has caused incompatibility between agricultural production and the rural environment, making the rural ecological environment increasingly severe [3,4]. Although agricultural production has been steadily increasing over the past few years, it has brought about problems such as excessive fertilizer inputs, nonpoint agricultural pollution, and hidden food safety problems, which have become a threat to human health and the living environment, and have seriously hindered the agricultural green development process [5,6,7]. Therefore, it is crucial for China to shift to the green development mode in order to protect and restore the cultivated land ecosystem, provide ecological agricultural products, and guarantee national food security at this stage.

In response to the existing environmental problems, the Chinese government has issued a series of policies to promote the wide application of green production techniques and realize the transformation of the agricultural mode. As farmers are the core of the agricultural production system, their behaviors affect green production’s effectiveness. Thus, it is necessary to shift farmers’ traditional ways to eco-friendly ones [8]. Under the government’s publicity and incentives, environmental protection and sustainable development are nothing new in China. Most Chinese farmers are willing to adopt green production techniques [9]. However, although most farmers have accepted and agreed to the value of ecological production, their traditional production behavior has not changed significantly. There is a certain degree of deviation between farmers’ green production willingness and their behavior, or there may be some obstacles to transforming farmers’ willingness into their behavior [10,11]. Hence, it is of great practical significance and theoretical value to study the mechanism underlying the deviation between farmers’ willingness to produce ecologically and their behavior.

Green production and pollution reduction in agriculture have been a global topic of broad and current interest [12,13]. Social behaviorists believe that individual will is the forerunner of behavior, and the two have a high degree of consistency [14,15,16]. In that case, why do farmers’ green production behaviors and willingness deviate from each other? So far, some achievements have been made in the analysis of farmers’ behavior from the perspective of disobedience between willingness and behavior, mainly focusing on food consumption, household waste disposal, environmental protection, and agricultural technology adoption [17]. Scholars have found that the factors affecting famers’ willingness and behavior are different, which can lead to deviations. Theoretically, the contradiction between farmer’s behavior and will is mainly the result of multiple factors, including individual characteristics and family endowment. For example, Li (2021) [18] analyzed the key factors that influence the willingness–behavior consistency of farmers to adopt photovoltaic agriculture, and Qiu (2022) [19] explored whether there is a deviation between farmers’ willingness and behavior to adopt electricity-saving tricycles, and they both concluded that gender, age, education, planting scale, and land transfer can significantly affect the deviation between farmers’ production behavior and willingness Guo (2021) [9] took rice farmers’ willingness and behaviors regarding the application of biopesticides as the research object, and argued that farmers’ green production cognition significantly negatively affects the deviation. Academics also found that the external environment—such as government support, regional differences, and proximity to cities—can affect farmers’ green production behavior–willingness deviation [20,21,22,23]. Moreover, the theory of planned behavior is often used to explain the factors that influence farmer’s behaviors and willingness [24]. 

The existing literature has provided a theoretical basis for this paper to study factors influencing the deviation between farmers’ green production willingness and behavior. However, there is still room for further exploration: first, relevant studies in agriculture are rare, with research topics being scattered widely. There are few empirical studies on farmers’ multiple green production behaviors and willingness. Most of the existing studies are based on a certain green production behavior, such as organic fertilizer application, pesticide reduction, or straw-returning technology adoption, etc. However, a single green production behavior cannot measure farmers’ overall green production application, and the research results may not be sufficient to guide farmers’ green production practices. Secondly, there are few studies on the degree of farmers’ green production behavior–willingness deviation. Previous studies have mainly focused on whether farmers’ behaviors and willingness deviate, but there is a lack of research on the degree of their deviation. Overall, there is still a gap in the research on the behavior–willingness deviation of green production, and influencing factors need to be analyzed. 

Based on the survey data of 436 households in Hainan Province, this paper selects three green production behaviors—namely, organic fertilizer application, soil testing adoption, and straw-returning—to measure the degree of deviation between farmers’ green production behaviors and willingness. On this basis, an ordered logit model was used to investigate the factors influencing the degree of deviation, and the ISM was used further to investigate the logical relationship structure between the influencing factors, in order to provide a more relevant reference basis for the formulation of relevant policies in other developing countries. Compared with the existing studies, the main contributions of this paper are: first, the influencing factors of farmers’ green production behavior–willingness deviation are identified, and the hierarchical structure of each influencing factor is further revealed, which has received less attention in the existing related studies; secondly, the adoption behavior of farmers for several green production technologies is included in the empirical analysis, such that the degree of deviation can be used as the explanatory variable, which makes the results of our empirical analysis more precise compared to existing studies; thirdly, using farmers in Hainan Province as the research object, we focus on the green development of agriculture in tropical China, and expand the regional scope of related research. 

## 2. Theoretical Framework

A combination of factors influences farmers’ willingness and behavior regarding green production, and this paper takes the rational smallholder theory as the primary theoretical basis, which believes that farmers are rational economic persons and their decision-making behavior is completely rational in a competitive market mechanism [25]. When there is a demand for green production, whether to take further action requires a comprehensive and rational consideration of various factors based on farmers’ willingness. In this paper, we focus on the main factors that influence the deviation of farmers’ green production behaviors and willingness, including individual characteristics, household characteristics, the external environment, and farmers’ green production cognition. The expected effects of each variable on farmers’ willingness–behavioral deviations are as follows. 

### 2.1. Farmers’ Individual Characteristics 

Individual characteristics mainly refer to the famer’s gender, age, ethnicity, education, health status, part-time employment, risk preference, information access, etc. In general, because men are more involved in agricultural practices as the main labor force, they have a better understanding of the actual situation of green production. They are more rational in making green production decisions, and tend to be more willing to try new production techniques, which motivates them to translate their willingness regarding green production into practical action [26,27]. Some scholars believe that older farmers have more experience and a higher knowledge of green production, and age significantly negatively affects farmers’ green production behavior–willingness deviation [28]. Meanwhile, some scholars argue that older farmers have certain deficiencies in cognitive ability and risk-taking ability, and as the application of green production technology has higher demands on labor, it is often difficult for older farmers to take practical actions [29,30]. It has also been proven that there are certain deviations in the implementation of green production between Han Chinese farmers and ethnic minority farmers, which may be due to the differences in topographical factors and traditional culture [31]. This is because ethnic minority farmers tend to live in areas closer to ecological reserves and have traditional notions of protecting the ecological environment, which will lead them to adopt more green production practices. Because education can reflect the cognitive ability of farmers, farmers with high education usually have higher cognitive ability and acceptance of green production; they have a deeper understanding of the economic and ecological benefits that green agricultural production can bring, so they are more likely to engage in green production [32,33]. The application of green production techniques requires a large amount of labor, and the health status of farmers is also a guarantee of labor input. Farmers with good health status are more likely to convert their willingness regarding green production into actual action, while farmers with poor health status may not be able to do so [34]. Farmers with part-time jobs will spend less time and labor on agricultural production, which may lead to a sloppy agricultural production pattern that is not conducive to green production [35]. Certain risks often accompany green production technologies, and those with a higher risk appetite are more willing to adopt green production technologies [36,37] actively; they may wish to reap the potential risk benefits of implementing green production practices. Farmers’ access to information can help them acquire knowledge and experience related to green production, and the more information famers obtain, the more they can deepen their knowledge of the benefits of green production, and the more they are likely to adopt green production [38,39]. In light of this, this research proposes Hypothesis 1: Farmers’ individual characteristics significantly affect their green production behavior–willingness deviation.

### 2.2. Farmers’ Household Characteristics

Household characteristics include the agricultural labor share, whether the family members are village cadres or civil servants, number of dependents, planting scale, land fragmentation, agricultural expenditure, and land transfer status. Scholars generally believe that households with a large proportion of agricultural labor are more likely to take agriculture as their primary source of income, thereby investing more time and energy in agricultural production, and paying more attention to the sustainability of agriculture [40]. Consequently, households with a larger share of agricultural labor are more likely to engage in green production [41]. Village cadres and civil servants can act as leaders, and are the driving force behind the popularization and diffusion of green production. Therefore, in order to play an exemplary role, they will be more active in adopting green production behaviors, which also reduces the degree of behavior–willingness deviation [42]. The number of dependents tends to influence farmers’ agricultural inputs [43]; farmers with more elderly dependents have less capital, human and material resources to invest in, which will prevent the farmers’ willingness regarding green production from being translated into actual behavior, such that it has a significant positive impact on farmers’ behavior–willingness deviation. Different scholars have reached inconsistent conclusions regarding the effects of planting scale and land fragmentation. It is usually believed that the larger the planting scale and the more fragmented the plots are, the higher the cost farmers have to spend and the lower the possibility of engaging in green production [44], while some scholars argue that the larger planting scale indicates that farmers rely mainly on agriculture as their source of income, and in order to ensure the quality of the land and the income sustainability, farmers tend to engage in green production [45,46]. Additionally, some studies have confirmed that although a higher number of plots may lead to more cost, farmers still tend to engage in green production when the benefits are more significant than the costs invested [47]. The increase in agricultural expenditure reflects the increased input and their dependence on agricultural production, such that farmers with high agricultural expenditure will pay more attention to the sustainable development of agriculture for higher returns in the long term, and their probability of green production will increase accordingly [48]. Farmers with land transfer tend to have larger land sizes, and are more likely to adopt green production to ensure land quality, such that land transfer has a significant negative effect on farmers’ green production behavior–willingness deviation [49]. In light of this, this research proposes Hypothesis 2: Farmers’ household characteristics significantly affect their green production behavior–willingness deviation.

### 2.3. External Environment 

The external environment refers mainly to peer influence, social networks, neighborhood learning, and agricultural extension services. Due to the unknown nature of agricultural production, farmers are vulnerable to the behavior of surrounding farmers when making decisions [50]. Still, there is often a great deal of uncertainty in the behavior of surrounding farmers, which may promote farmers toward green production, or may make them maintain their original rough production patterns. Therefore, peer effect significantly impacts farmers’ green production behavior–willingness deviation, but the direction of the impact is uncertain. Generally, extensive social networks can help farmers to obtain more information and have a better understanding of the green production mode and green grains, thereby promoting their green production behavior [29,51], while sometimes, the bias and distortion of information obtained through a social network can also make farmers reluctant to engage in green production. Agricultural communication and learning among neighbors can help to improve farmers’ awareness of environmental protection and green production perceptions, which can facilitate the conversion of green production intentions to behaviors [52]. Agricultural extension services can increase the motivation of farmers to engage in green production and raise farmers’ green production awareness and technology level, which in turn promotes green production [53,54]. Given this, this research proposes Hypothesis 3: The external environment significantly affects farmers’ green production behavior–willingness deviation.

### 2.4. Green Production Cognition

Green production cognition mainly includes environmental pollution cognition, economic development cognition, government policy understanding, and ecological knowledge. Studies have shown that farmers’ behavior is often determined by their cognitive ability [55,56,57]. The stronger the farmers’ green production cognitions, the higher the probability of converting green production intentions into actual behaviors. Farmers’ environmental perception ability—their degree of understanding of reality, policies, and ecological knowledge—can facilitate the conversion of their green production intentions into behaviors. In light of this, this research proposes Hypothesis 4: Farmers’ green production cognition significantly affects their green production behavior–willingness deviation.

**Figure 1:**
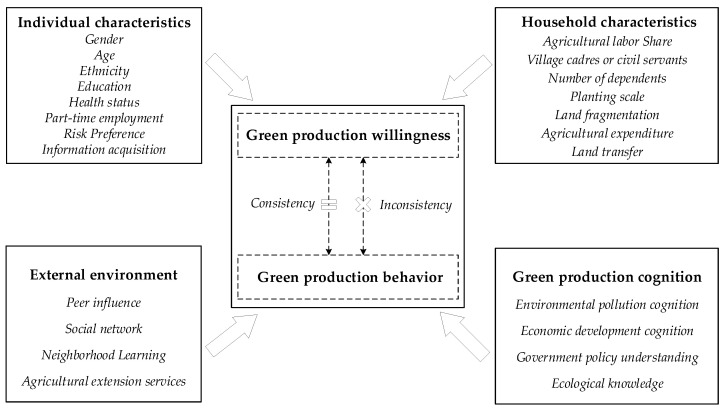
The conceptual framework.

Figure 1 shows the conceptual framework of factors influencing farmers’ agricultural green production behavior-willingness deviation.

## 3. Data and Methods

### 3.1. Data Source

The study area of this paper is Hainan Province, the southernmost province in China. Against the strategic background of national ecological civilization construction, Hainan Province’s agriculture urgently needs to change from traditional operation patterns to environmentally friendly ones. We chose Hainan province as the study area mainly based on the following reasons: Hainan Island is separated from mainland China by the Qiongzhou Strait, the transportation cost of agricultural products is higher, and coupled with climate and environmental factors, Hainan farmers mostly grow high-value agricultural products such as mangoes, natural rubber, bananas, cocoa, and other tropical crops, which requires higher inputs of chemical fertilizers and pesticides. This feature of Hainan’s agricultural production makes it different from the production of field crops in mainland China, and it is also essential to promote the concept and technology of green agricultural production for Hainan farmers. However, there is a relative lack of research on the willingness or behavior of green agricultural production in tropical China.

Research data were obtained from a field survey conducted from July to August 2021. Multistage stratified sampling and random sampling methods were used to select the samples. Firstly, four cities, namely Haikou, Dongfang, Qiongzhong, and Lingshui, were purposively selected with a comprehensive consideration of representativeness, economic development, and geographical location. In the second stage, 2~4 towns were randomly selected from each county, and 4~5 villages were randomly selected in each town. Finally, 20~30 farmers were randomly selected from each village. The questionnaire mainly included the individual characteristics of the farmers, household characteristics, farmers’ green production behavior and willingness, information access, and ecological awareness, etc. In order to ensure the quality and validity of the survey data, our research team conducted very detailed training for all of the interviewers. Before filling out the questionnaire formally, the interviewer explained the purpose of the survey and the operation process in detail to the interviewees in order to ensure the authenticity and validity of their responses. All of the surveys were conducted through face-to-face interviews. Finally, 666 valid samples were obtained, with an effective rate of 97.94%. Because we aimed to analyze the main influencing factors for behavior–willingness deviation, after screening and excluding the farmers without green production intention, a total of 436 samples were used for analysis.

Table 1 shows the socio-demographic characteristics of the respondents. Male farmers account for 93.81% of the sample, as we selected household heads who are more aware of their household production for interviews. The sample farmers are mainly middle-aged and older, with the largest proportion being 41–50 years old, accounting for 30.50% of the total sample. Han Chinese and ethnic minorities account for 66.97% and 33.03% of the sample farmers, respectively. The sample farmers are generally low-educated, with 48.17% being junior high school graduates. The planting scale of the farmers interviewed is mainly less than ten mu, accounting for 65.83% of the total sample. 36.93% of the farmers’ agricultural expenditure is less than CNY5000, while farmers with more than CNY20,000 account for 20.18%. In total, 32.57% of the sample farmers’ households have village cadres or civil servants, and 41.97% of the farmers have part-time jobs.

### 3.2. Variable Settings

#### 3.2.1. Explained Variable

According to the theoretical framework, this paper defines farmers’ green production behavior–willingness deviation as the explained variable. We measured the degree of inconsistency between farmer’s willingness and behavior in three aspects—namely the application of organic fertilizer, the adoption of soil testing and formula fertilization technology, and the application of straw-returning technology—to analyze farmers’ green production behavior–willingness deviation. According to the theory of planned behavior, willingness is a prerequisite for behavior, and willingness determines behavior generation. In this paper, we quantify and assign values to three types of behaviors and the degree of deviation from willingness. If all three behaviours and willingness of the farmers’ are consistent, i.e., there is no deviation between the behaviors and willingness, the value is 0. Suppose there is a green production technology that farmers are willing to adopt but not actually adopt, a low deviation is assigned, and there are two. In that case, it is assigned as medium deviation. If all three technology adoption behaviors of the farmers deviate from their willingness, a high deviation is assigned. The statistics of the farmers’ green production behavior–willingness deviation are shown in Table 2.

#### 3.2.2. Explanatory Variable

This paper selected individual characteristics, household characteristics, the external environment, and green production cognition as explanatory variables based on existing studies. Individual characteristics include farmers’ gender, age, ethnicity, education, health status, part-time employment, and risk preference [58,59,60,61]. Household characteristics include the agricultural labor share, number of dependents, planting scale, land fragmentation, agricultural expenditure, and land transfer. The external environment refers to peer influence, village cadres or civil servants, information acquisition, social networks, neighborhood learning, and agricultural extension services. Green production cognition includes farmers’ environmental pollution cognition, economic development cognition, government policy understanding, and ecological knowledge. The definition of the variables and their descriptive statistics are shown in Table 3.

### 3.3. Model Selection

#### 3.3.1. Ordered Logit Model

The degree of farmers’ green production behavior–willingness deviation is an ordered categorical variable, and this paper adopts a multiple-ordered regression equation for empirical analysis. The specific model expression is as follows:

(1) $$Ln\left(\frac{p_{j}}{1-p_{j}}\right)=\alpha_{j}+\sum_{i=1}^{n}\beta_{i}x_{i}$$

where $p_{j}=p(y\leq j|x)$ denotes the cumulative probability of the dependent variable taking the first j values, and the cumulative probability function is expressed as

(2) $$P_{i}=p\left(y\leq j|x\right)=\frac{\exp\left(\alpha_{j}+\sum_{i=1}^{n}\beta_{i}x_{i}\right)}{1+\exp\left(\alpha_{j}+\sum_{i=1}^{n}\beta_{i}x_{i}\right)}$$

where $p_{j}\;$denotes the probability that farmers’ behavior deviates from their willingness, $\alpha_{j}$ denotes the intercept parameter, $\beta_{i}$ denotes the regression coefficients of different influencing factors, and $x_{i}$ denotes individual characteristics, household characteristics, external environment, and green production cognition.

#### 3.3.2. ISM Model

**Figure 2:**
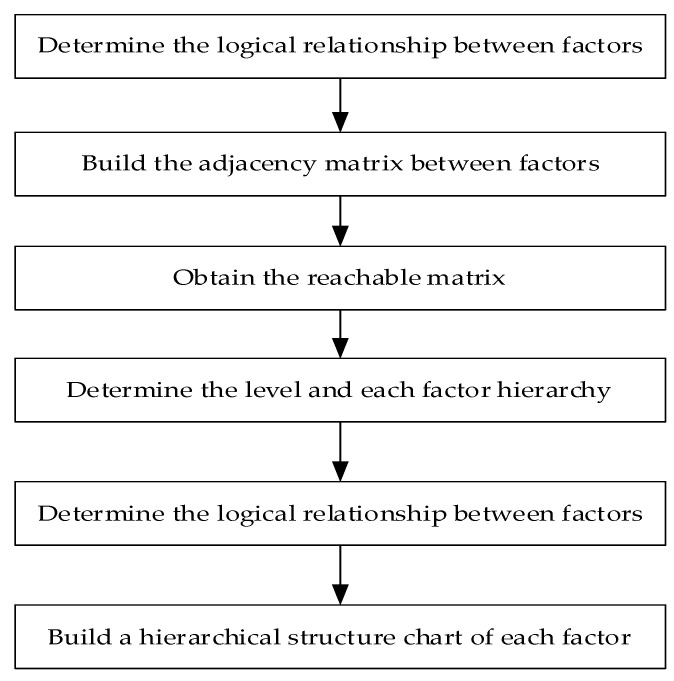
The steps of the ISM.

The factors influencing the deviation between famers’ willingness to adopt green production and their behavior are independent and interrelated; it is crucial to distinguish the hierarchy of relationships among factors in order to identify the key reasons for behavior–willingness deviation. Therefore, this paper further analyzes the correlation and hierarchy between factors influencing the deviation using the ISM model. The steps of the ISM are shown in Figure 2.

It is assumed that there are k factors that influence farmers’ behavior–willingness deviation obtained using the multivariate ordered logit model; *S*_0_ denotes the behavior–willingness deviation, *S_i_* (*i =* 1, 2, 3…, *k*) denotes the influencing factors, and each constituent factor in the adjacency matrix R is determined according to Equation (3).

(3) $$R_{ij}=\left\{\begin{array}{c} 1,\;\;S_{i}\;is\;related\;to\;S_{j}{}_{}\\ \;\;\;\;0,\;\;S_{i}\;is\;not\;related\;to\;S_{j}{}_{} \end{array}\right\}\;\;i,\;j=1,\;2\cdots ,\;k\;$$

The reachable matrix of factors influencing farmers’ behavior–willingness deviation can be expressed as

(4) $$\;\;\;\;\;\;M={\left(R+I\right)}^{\lambda +1}={\left(R+I\right)}^{\lambda }\neq {\left(R+I\right)}^{\lambda -1}\neq {\left(R+I\right)}^{\lambda -2}\cdots \neq {\left(R+I\right)}^{2}\neq \left(R+I\right)$$

where: 2 ≤ λ ≤ *k*, *I* represent the unit matrix of the factors influencing farmers’ behavior–willingness deviation, and the power operation is applied to the unit matrix using the Boolean operator.

The hierarchical structure between the influencing factors can be determined according to Equation (5):

(5) $$\;L=\left\{S_{i}|P\left(S_{i}\right)\cap Q\left(S_{i}\right)=P\left(S_{i}\right)\right\}\;\left(i,\;\;j=1,\;2\cdots ,\;k\right)$$

where $P\left(S_{i}\right)$ is the reachable set of influencing factors of farmers’ behavior–willingness deviation, which indicates the set of all factors that can be reached from the influencing factor *S_i_*. First, the influence factors contained in the highest level *L_i_* can be measured using Equation (5). The other levels and the influence factors contained in the levels are determined sequentially according to the same method. Secondly, each level is reordered to obtain a new reachable matrix T. Finally, each level of factors influencing farmers’ behavior–willingness deviation is connected using directed edges to obtain a hierarchy diagram.

## 4. Results

### 4.1. Analysis of Factors Influencing Farmers’ Behavior–Willingness Deviation

Stata15.1 (Version 15.1, created by StataCorp LLC., College Station, TX, USA) software was used for the model estimation. The variables were tested for multicollinearity before the regression analysis in order to circumvent multicollinearity. The results showed that the variance inflation factors (VIF) were all less than 5, indicating that there was no problem with multicollinearity. Table 4 shows the estimated results.

#### 4.1.1. Effect of Individuals’ Characteristics

As seen from the regression results in Table 4, age has a significant positive effect on farmers’ green production behavior–willingness deviation, which is significant at the 10% level, indicating that older farmers’ green production behavior is more likely to deviate from their willingness, which is consistent with the study of Xu (2021) et al. [60]. The possible reason for this is that the implementation of green production and the adoption of green production technologies both place higher requirements on farmers’ cognitive abilities and labor inputs, such that even if older farmers can recognize the benefits of green production, it is difficult for them to put them into action, resulting in a deviation between their behavior and willingness. The estimated coefficient of ethnicity is negative and significant at the 10% level, indicating that the probability of behavior–willingness deviation is lower for Han Chinese farmers than ethnic minorities. This may be because ethnic areas are mostly mountainous and geographically influenced, which makes applying green production difficult and thus leads to deviation. The education level is significant at the 10% level with a negative sign, indicating that higher education-level farmers are more likely to convert their green production intentions to behavior. Education level can reflect the cognitive level and acceptability of farmers, and the more educated farmers are, the stronger their awareness of green production and the more willing they are to convert their willingness into behavior. The estimated coefficients of gender, health status, part-time employment, risk preference, and information acquisition are insignificant, indicating that the above variables have no significant effect on farmers’ behavior–willingness deviation.

#### 4.1.2. Effect of Household Characteristics

The number of dependents is significant at the 10% level. The estimated coefficient is positive, indicating that the family with more dependents is more likely to deviate the green production behavior from willingness, mainly because adopting green production technology requires a large capital investment. When the household has more older people to support in their daily consumption and care, the family is less likely to have extra funds for green production, reducing the conversion of green production intentions to behaviors. Land fragmentation is significant at the 10% level with a negative sign, indicating that the higher the degree of land fragmentation, the more farmers’ green production behavior and willingness tend to be consistent. This is inconsistent with most of the previous studies. The possible reason is that although the farmers have more plots, they will still adopt green production technologies when the benefits are higher than the extra costs due having to more plots. Agricultural expenditure is negatively significant at the 5% level, indicating that the more agricultural expenditure there is, the less likely farmers’ behavior and willingness regarding green production are to deviate. The reason may be that the increase in agricultural expenditure indicates that farmers have more inputs in agricultural production, and the demand for sustainable agricultural development is increasing, such that the probability of farmers engaging in green production increases accordingly. Land transfer has a significant negative effect on the deviation at the 10% level, indicating that farmers who have transferred land are less likely to deviate in their green production behavior from their willingness, probably because land transfer promotes large-scale operation and reduces the cost of adopting green production technologies, which in turn promotes the adoption of green production technologies. The estimated coefficients of the agricultural labor share, village cadres or civil servants and farming scale are insignificant, indicating that they have no significant effect on farmers’ deviation of green production behavior and willingness.

#### 4.1.3. Effect of the External Environment

Regarding the external environment, peer influence is significant at the 5% level with a positive sign, indicating that farmers with stronger peer influence have a higher probability of deviating their green production behavior from intentions. This may be due to the nature of farmers’ limited cognition, which tends to follow the behavior of surrounding farmers when making decisions, and when most farmers’ neighbors or relatives are not practicing green production, they will follow other farmers, thereby leading to behavior–willingness deviation. Social networks are positively significant at the 10% level, indicating that the more extensive the social network is, the more likely the farmers’ green production behavior and willingness to deviate from each other, which is probably because information obtained from social networks block their progress towards greener production. Neighborhood learning is significant at the 1% level and the estimated coefficient is negative, indicating that mutual learning among neighbors significantly affects the deviation. The possible reason is that farmers’ frequent agricultural production communication with surrounding farmers may increase the level of knowledge and awareness about green production, which in turn promotes farmers’ willingness to transform into practical actions. The effect of agricultural extension services were not significant.

#### 4.1.4. Effect of Green Production Cognition

As for farmers’ green production cognition, both environmental pollution cognition and ecological knowledge significantly affect the deviation. The negative effect of environmental pollution cognition at the 10% level indicates that the stronger farmers’ knowledge of environmental pollution, the less likely their green production behavior and willingness are to deviate from each other, and the negative estimated coefficient of ecological knowledge at the 1% level indicates that the more farmers know about ecological knowledge, the more they can inhibit the green production behavior–willingness deviation. This may be because farmers’ knowledge of ecology can help them understand the economic and ecological benefits that green production can bring, and can convert their willingness into practical actions. The estimated coefficients of economic development cognition and government policy understanding did not pass the significance test.

#### 4.1.5. Marginal Effect Analysis

Because the economic meaning of the coefficient terms estimated is not intuitive, it only contains information on the statistical significance and influence direction of the explanatory variables. In order to further obtain the degree of influence of each explanatory variable, we calculated the marginal effects of the explanatory variables. To save space, we report only the marginal effects of the significant variables (according to Table 4), as shown in Table 5.

From the analysis of the marginal effects, the meaning of the estimated coefficient is the probability that for each unit change in the explanatory variable, the probability that the farmer’s behavior deviates from their intentions increases (if the estimated coefficient is positive) or decreases (if the estimated coefficient is negative). For example, when farmers’ age increases by one unit, the probability of green production behavior deviating from their willingness decreases by 0.003 for “slight deviation”, increases by 0.002 for “medium deviation”, and increases by 0.002 for “high deviation”. Among the significant variables, we find that farmers’ ecological knowledge has the largest effect on farmers’ behavior–willingness deviation, followed by ethnicity and land transfer, which provides interesting inspirations for us when designing promotion measures.

### 4.2. ISM Analysis Results

**Figure 3:**
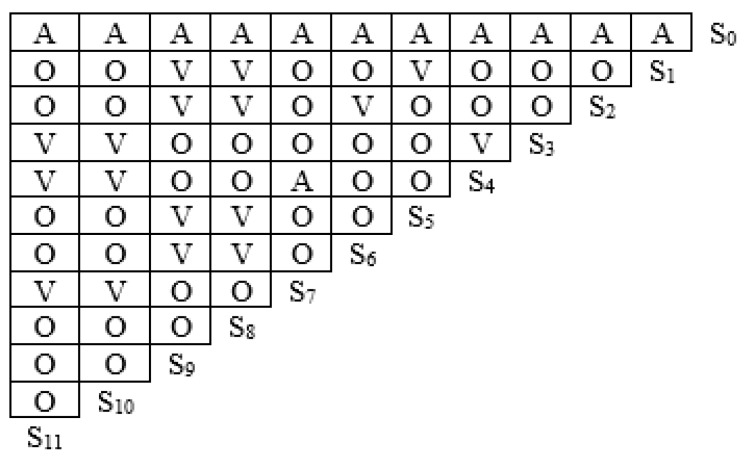
Logical relationship diagram of the influencing factors.

From the previous regression results, it can be seen that the factors influencing farmers’ green production behavior–willingnes deviation are age, ethnicity, education, neighborhood learning, number of dependents, land fragmentation, social network, agricultural expenditure, land transfer, peer influence, and green production cognition. Therefore, this paper characterizes them by *S_i_* (*i =* 1, 2, 3…, 11), respectively, and *S*_0_ denotes the green production behavior and willingness to deviate. Based on theoretical analysis and expert consultation, the logical relationships between the factors were determined as shown in Figure 3, where V indicates that the row factor has a direct or indirect influence on the column factor, A indicates that the column factor has a direct or indirect influence on the row factor, and O indicates that there is no mutual influence between the row and column factors.

According to the logical relationship between the influencing factors in Figure 3 and Equation (3), we can obtain the adjacency matrix R between each influence factor:

$$R=\begin{array}{c} \begin{array}{c} \begin{array}{c} \begin{array}{c} \begin{array}{c} \begin{array}{c} S_{0}\\ S_{1} \end{array}\\ S_{2} \end{array}\\ S_{3} \end{array}\\ S_{4} \end{array}\\ S_{5}\\ S_{6} \end{array}\\ S_{7}\\ S_{8}\\ \begin{array}{l} S_{9}\\ S_{10}\\ S_{11} \end{array} \end{array}\left[\begin{array}{lllllllllllll} 0 & 0 & 0 & 0 & 0 & 0 & 0 & 0 & 0 & 0 & 0 & 0\\ 1 & 0 & 0 & 0 & 0 & 1 & 0 & 0 & 1 & 1 & 0 & 0 & \\ 1 & 0 & 0 & 0 & 0 & 0 & 1 & 0 & 1 & 1 & 0 & 0\\ 1 & 0 & 0 & 0 & 1 & 0 & 0 & 0 & 0 & 0 & 1 & 1\\ 1 & 0 & 0 & 0 & 0 & 0 & 0 & 0 & 0 & 0 & 1 & 1\\ 1 & 0 & 0 & 0 & 0 & 0 & 0 & 0 & 1 & 1 & 0 & 0\\ 1 & 0 & 0 & 0 & 0 & 0 & 0 & 0 & 1 & 1 & 0 & 0\\ 1 & 0 & 0 & 0 & 1 & 0 & 0 & 0 & 0 & 0 & 1 & 1 & \\ 1 & 0 & 0 & 0 & 0 & 0 & 0 & 0 & 0 & 0 & 0 & 0\\ 1 & 0 & 0 & 0 & 0 & 0 & 0 & 0 & 0 & 0 & 0 & 0\\ 1 & 0 & 0 & 0 & 0 & 0 & 0 & 0 & 0 & 0 & 0 & 0\\ 1 & 0 & 0 & 0 & 0 & 0 & 0 & 0 & 0 & 0 & 0 & 0 \end{array}\right]$$

Using Matlab (Version R2021b, created by The MathWorks in Natick, MA, USA) software and Equation (4), the reachable matrix M is further calculated from the adjacency matrix R, as follows:

$$M=\begin{array}{c} \begin{array}{c} \begin{array}{c} \begin{array}{c} \begin{array}{c} \begin{array}{c} S_{0}\\ S_{1} \end{array}\\ S_{2} \end{array}\\ S_{3} \end{array}\\ S_{4} \end{array}\\ S_{5}\\ S_{6} \end{array}\\ S_{7}\\ S_{8}\\ \begin{array}{l} S_{9}\\ S_{10}\\ S_{11} \end{array} \end{array}\left[\begin{array}{lllllllllllll} 1 & 0 & 0 & 0 & 0 & 0 & 0 & 0 & 0 & 0 & 0 & 0\\ 1 & 1 & 0 & 0 & 0 & 1 & 0 & 0 & 1 & 1 & 0 & 0\\ 1 & 0 & 1 & 0 & 0 & 0 & 1 & 0 & 1 & 1 & 0 & 0\\ 1 & 0 & 0 & 1 & 1 & 0 & 0 & 0 & 0 & 0 & 1 & 1\\ 1 & 0 & 0 & 0 & 1 & 0 & 0 & 0 & 0 & 0 & 1 & 1\\ 1 & 0 & 0 & 0 & 0 & 1 & 0 & 0 & 1 & 1 & 0 & 0\\ 1 & 0 & 0 & 0 & 0 & 0 & 1 & 0 & 1 & 1 & 0 & 0\\ 1 & 0 & 0 & 0 & 1 & 0 & 0 & 1 & 0 & 0 & 1 & 1 & \\ 1 & 0 & 0 & 0 & 0 & 0 & 0 & 0 & 1 & 0 & 0 & 0\\ 1 & 0 & 0 & 0 & 0 & 0 & 0 & 0 & 0 & 1 & 0 & 0 & \\ 1 & 0 & 0 & 0 & 0 & 0 & 0 & 0 & 0 & 0 & 1 & 0\\ 1 & 0 & 0 & 0 & 0 & 0 & 0 & 0 & 0 & 0 & 0 & 1 \end{array}\right]$$

**Figure 4:**
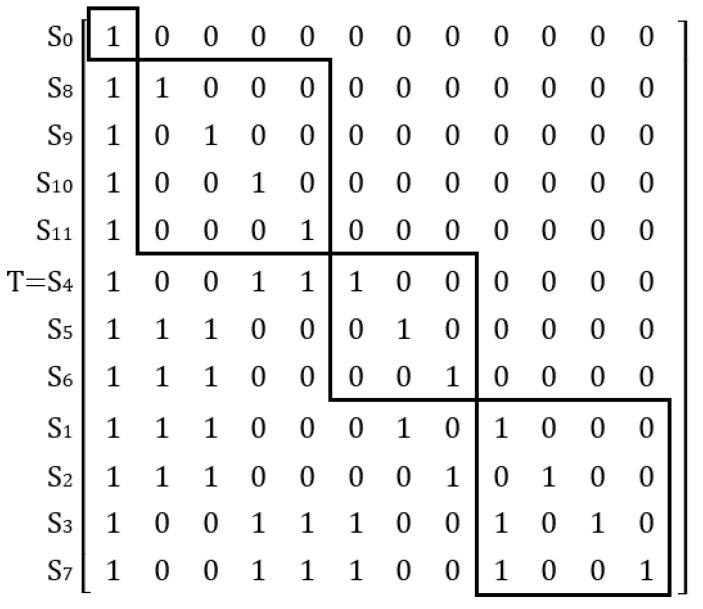
Driving factor hierarchy T diagram.

Finally, *L*_1_ = {*S*_0_} is obtained based on the determination of the highest-level factor. Similarly, based on the same method, we obtain *L*_2_ = {*S*_8_, *S*_9_, *S*_10_, *S*_11_}, *L*_3_ = {*S*_4_, *S*_5_, *S*_6_}, *L*_4_ = {*S*_1_, *S*_2_, *S*_3_, *S*_4_}. The reachability matrix is re-measured according to the above hierarchy, and a hierarchy T diagram of factors influencing farmers’ behavior–willingness deviation can be obtained as shown in Figure 4.

**Figure 5:**
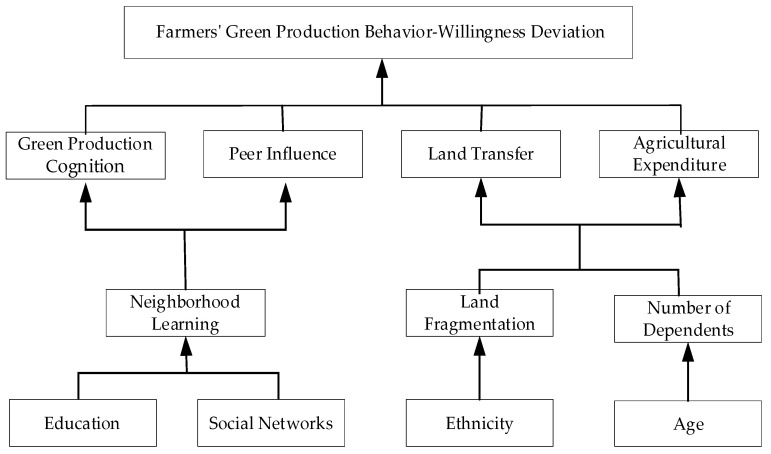
Interpretative structural model of the influencing factors.

According to the hierarchy of factors influencing farmers’ green production behavior–willingness deviation, it can be seen that farmers’ behavior–willingness deviation is at the first level. Green production cognition, peer influence, land transfer, and agricultural expenditures are in the second level. Neighborhood learning, land fractionalization, and the number of dependents are in the third level. Education, social network, age, and ethnicity are at the bottom of the hierarchy. The influencing factors of adjacent levels are connected with directed arrows, and finally, the explanatory structure model of each influencing factor is obtained. As shown in Figure 5, there are three main paths. Path ①: farmers’ education, social network → neighborhood learning → green production cognition, peer influence → green production behavior–willingness deviation. Path ②: ethnicity → land fragmentation → land transfer, agricultural expenditure → green production behavior–willingness deviation. Path ③: age → number of dependents → land transfer, agricultural expenditure → green production behavior–willingness deviation. Green production cognition, peer influence, land transfer, and agricultural expenditure are the most direct factors influencing farmers’ behavior–willingness deviation. Neighborhood learning, land fragmentation, and the number of dependents are indirect factors that influence farmers’ behavior–willingness deviation. Educational level, social network, age, and ethnicity are the most fundamental factors influencing farmers’ behavior–willingness deviation.

## 5. Discussion and Conclusions

It is of great significance for farmers to carry out green production to promote green and high-quality agricultural development, and to realize rural ecological revitalization. Based on the field research data in Hainan Province, this paper empirically analyzes the factors influencing farmers’ green production behavior–willingness deviation, and explores the logical hierarchy among the influencing factors. The main findings are as follows: firstly, 94.72% of the farmers’ green production behavior and willingness are not consistent; there are deviations between the farmers’ green production behavior and willingness, which is consistent with the results obtained by Guo et al. (2021) [9], Liu et al. (2021) [17] and Xu et al. (2021) [60], indicating that there is also a serious behavior–willingness deviation in the process of green agricultural production in tropical China. Secondly, age, number of dependents, peer influence, and social network have a significant positive effect on farmers’ green production behavior–willingness deviation, while ethnicity, education, land fragmentation, agricultural expenditure, land transfer, neighborhood learning, and green production cognition have a negative influence on the deviation. Finally, among the significant influencing factors, farmers’ perceptions of green production, peer influence, land transfer, and agricultural expenditure are the surface direct factors; neighborhood learning, land fragmentation, and the number of dependents are the middle indirect factors; and farmers’ education, social network, age, and ethnicity are the deep-rooted factors. The significance of the above influencing factors and their hierarchical relationships are somewhat different from existing studies, probably due to the differences in agricultural production and the individual characteristics of farmers in the tropics and inland. The results of these differences create more practical and relevant guidance for the green development of agriculture in tropical areas of China.

Based on the findings of this paper, we propose the following policy recommendations: first, increase the publicity and education on green production in order to improve farmers’ knowledge of green production. On the one hand, more farmers can see green production’s ecological benefits and business gains through face-to-face lectures and field demonstrations. At the same time, we should play up the role of neighborhood learning and the peer effect on the surrounding farmers’ green production; we should set up typical examples of propaganda and demonstration, and give substantial encouragement to guide more farmers in green production. Secondly, with the massive popularity of smartphones among farmers’ groups, new media has gradually become one of the essential platforms for propaganda and education. Therefore, we should apply new media in policy propaganda, and we should demonstrate the effect of green production technologies to farmers, using TV, WeChat, bulletin boards and other new media to promote their green production behavior. Third, we found that the age of farmers positively affects the deviation of farmers’ willingness regarding green production behavior. That is, the older the farmers are, the more deviant their willingness regarding green agricultural production behavior is. Therefore, for the relatively older farmers, we should carry out targeted and regular green production promotion and guidance work, and even send professional technicians into the countryside and villages to provide one-on-one guidance and assistance.

In contrast to previous studies on the factors influencing the deviation of farmers’ green production behaviors and intentions, this paper focuses not only on the “deviation or not” but also on the degree of deviation between behaviors and willingness [62]. Moreover, our study focuses on farmers’ adoption behavior and willingness regarding organic fertilizer application technology, soil testing technology, and straw-returning technology. In contrast, previous studies have mostly focused on one type of green production behavior. Secondly, this paper further focuses on the deeper relationship between the influencing factors, which is rarely seen in the existing studies on farmers’ behavior–willingness deviation. In summary, the main contribution of this paper is that this study is generalizable and accurate in its findings, and it also provides a more in-depth and detailed understanding of the factors influencing farmers’ green production behavior–willingness deviation.

In addition, there are also some limitations in the paper. First, due to the inevitable complexity of the agricultural production process, farmers may adopt multiple green production technologies and take multiple green production actions, and there are certain linkages between various behaviors. For example, when constrained by production costs, adopting one green production technology may affect the adoption of another, which needs to be considered in the farmers’ production decision process. Therefore, in future research, the interrelationship between different green production behaviors, and the way in which this interrelationship affects the deviation of farmers’ willingness regarding green production behaviors can be further explored. Secondly, this paper explores the reasons for the deviation of farmers’ green production behaviors and intentions from their perspective, which is a more subjective perspective. However, in actual production, the reasons for the deviation between green production behavior and willingness may be diverse, including many objective factors, such as some attributes of green production technology. In future studies, factors such as the ease of use, effectiveness, and cost–benefit of green production technologies can be further considered and incorporated into the research framework. In addition, the paper only explores farmers’ green production behavior and willingness at one point. The relationship between the two may change as the policy publicity increases and the farmers’ cognitive level continues to improve. Therefore, in future research, tracking survey data can be further used to explore the changes i\n farmers’ willingness regarding green production behavior before and after the policy implementation, or before and after the key time points to test the policy implementation effects.

## Figures and Tables

**Table 1:** Descriptive statistics of the sample farmers.

Items	Levels	Obs.	Frequency (%)	Items	Levels	Obs.	Frequency (%)
Gender	Male	409	93.81	Education (years)	≤6	108	24.77
	Female	27	6.19		7~9	210	48.17
Age (years)	21–30	19	4.36		10~12	89	20.41
	31–40	92	21.10		≥13	29	6.65
	41–50	133	30.50	Planting scale(mu)	<5	132	30.28
	51–60	125	28.67		5–10	155	35.55
	>60	67	15.37		11–20	80	18.35
Ethnicity	Han	292	66.97		>20	69	15.82
	Minority	144	33.03	Agricultural expenditure (one thousand yuan)	<1	20	4.59
Village cadres or civil servants	Yes	142	32.57		1–5	141	32.34
	No	294	67.43		5–10	104	23.85
Part-time employment	Yes	183	41.97		10–20	83	19.04
	No	253	58.03		>20	88	20.18

**Table 2:** The statistics of farmers’ green production behavior–willingness deviation.

Dependent Variable	Variable Interpretation	Options	Sample Size	Percentage (%)
Green production behavior	Is organic fertilizer used?	Used	242	55.50
		Not used	194	44.50
	Is soil testing technology adopted?	Used	276	63.30
		Not used	160	36.70
	Is straw-returning technology adopted?	Used	54	12.39
		Not used	382	87.61
Behavior–willingness deviation	The degree of farmers’ green production behavior–willingness deviation	No deviation	23	5.28
		Slight deviation	145	33.26
		Medium deviation	214	49.08
		High deviation	54	12.38

**Table 3:** Variables of the model and descriptive statistics.

Items	Variables	Definition	Mean	S. D.	Min	Max	Expected Direction
Dependent Variable	Behavior–willingness deviation	0 = No deviation; 1 = Slight deviation; 2 = Medium deviation; 3 = High deviation	1.68	0.75	0	3	—
Individual characteristic variables	Gender	1 = Male; 0 = Female	0.94	0.24	0	1	−
	Age	Respondent’s age (years)	49.21	11.15	23	78	?
	Ethnicity	1 = Han; 0 = Minority	0.67	0.47	0	1	−
	Education	0 = Never attended school; 1 = Primary school; 2 = Middle school; 3 = High school; 4 = College and above	2.04	0.93	0	4	−
	Health status	0 = Unable to care for themselves; 1 = Unable to do heavy work; 2 = Completely healthy	1.86	0.38	0	2	−
	Part-time employment	Whether the respondent has a part-time job. 1 = Yes; 0 = No	0.42	0.49	0	1	+
	Risk Preference	Do you think you are an adventurous person? 1 = Strongly disagree, 2 = Disagree, 3 = Neither agree nor disagree, 4 = Agree, 5 = Strongly agree	2.25	1.45	1	5	−
	Information acquisition	Whether the respondent has access to helpful information via the internet 1 = Yes; 0 = No	0.50	0.50	0	1	−
Household characteristic variables	Agricultural labor share	Number of farm laborers/number of family laborers	0.88	0.22	0.14	1	−
	Village cadres or civil servants	Whether there are village cadres or civil servants among the family members. 1 = Yes; 0 = No	0.33	0.47	0	1	−
	Number of dependents	Number of people in households who are not able to work	0.71	0.89	0	5	+
	Planting scale	Area of land operated by respondents	15.40	28.64	0.5	310	?
	Land fragmentation	Number of parcels of land owned by respondents	5.80	4.47	1	40	−
	Agricultural expenditure	The total annual expenses of respondents’ households	2.60	8.39	0.034	110	−
	Land transfer	Whether to rent or rent out farmland. 1 = Yes; 0 = No	0.33	0.47	0	1	−
External environment variables	Peer influence	Does the farming behavior of the surrounding farmers affect you? 1 = Rare, 2 = Few, 3 = Fair, 4 = More, 5 = Very much	2.46	1.56	1	5	?
	Social network	Households’ annual spending on personal relationships.	0.51	0.87	0	8	?
	Neighborhood Learning	How often do you communicate with people in your village to discuss agricultural production? 1 = Rare, 2 = Few, 3 = Fair, 4 = More, 5 = Very much	3.82	1.37	1	5	−
	Agricultural extension services	Whether the respondent has received agricultural extension services. 1 = Yes; 0 = No	0.65	0.48	0	1	−
Green production cognition variables	Environmental pollution cognition	Do you feel that the local ecological environment has deteriorated in recent years? 1 = Strongly disagree, 2 = Disagree, 3 = Neither agree nor disagree, 4 = Agree, 5 = Strongly agree	2.79	1.63	1	5	−
	Economic development cognition	Do you feel that protecting the environment will limit local economic development 1 = Strongly disagree, 2 = Disagree, 3 = Neither agree nor disagree, 4 = Agree, 5 = Strongly agree	2.29	1.54	1	5	−
	Government policy understanding	Do you understand the phrase “lucid waters and lush mountains are invaluable assets”. 0 = No; 1 = Fairly; 2 = Very	0.85	0.82	0	2	−
	Ecological knowledge	Do you understand the word “ecology”. 0 = No; 1 = Fairly; 2 = Very	0.73	0.81	0	2	−

**Table 4:** Regression results of the ordered logit model.

Variable Type	Variable Name	Regression Coefficient	Inspection Error S. E.	Z-Value
Individual characteristic variables	Gender	0.335	0.396	0.85
	Age	0.019 *	0.011	1.69
	Ethnicity	−0.401 *	0.213	−1.88
	Education	−0.197 *	0.118	−1.68
	Health status	0.201	0.252	0.80
	Part-time employment	−0.009	0.205	−0.04
	Risk Preference	−0.052	0.068	−0.76
	Information acquisition	0.183	0.243	0.75
Household characteristic variables	Agricultural labor share	0.541	0.455	1.19
	Village cadres or civil servants	0.122	0.219	0.55
	Number of dependents	0.213 *	0.111	1.92
	Planting scale	0.003	0.006	0.42
	Land fragmentation	−0.039 *	0.022	−1.72
	Agricultural expenditure	−0.047 **	0.022	−2.10
	Land transfer	−0.362 *	0.209	−1.74
External environment variables	Peer influence	0.136 **	0.064	2.15
	Social network	0.201 *	0.108	1.86
	Neighborhood Learning	−0.251 ***	0.074	−3.38
	Agricultural extension services	−0.156	0.212	−0.73
Green production cognition variables	Environmental pollution cognition	−0.104 *	0.058	−1.77
	Economic development cognition	−0.013	0.064	−0.21
	Government policy understanding	0.232	0.148	1.57
	Ecological knowledge	−0.473 ***	0.150	−3.14
	Sample size	436		

***, **, and * indicate 1%, 5%, and 10% significance levels, respectively.

**Table 5:** Marginal effect analysis results.

Variable Type	Variable Name	No Deviation	Slight Deviation	Medium Deviation	High Deviation
Individual characteristic variables	Age	−0.001	−0.003 *	0.002 *	0.002 *
	Ethnicity	0.018 *	0.066 *	−0.044 *	−0.041 *
	Education	0.009	0.033 *	−0.022 *	−0.020 *
Household characteristic variables	Number of dependents	−0.010 *	−0.035 *	0.023 *	0.022 *
	Land fragmentation	0.002 *	0.006 *	−0.004 *	−0.004 *
	Agricultural expenditure	0.002 **	0.008 **	−0.005 **	−0.005 **
	Land transfer	0.016	0.060 *	−0.040 *	−0.037 *
External environment variables	Peer influence	−0.006 **	−0.023 **	0.015 **	0.014 **
	Social network	−0.009 *	−0.033 *	0.022 *	0.020 *
	Neighborhood Learning	0.011 ***	0.041 ***	−0.027 ***	−0.025 ***
Green production cognition variables	Environmental pollution cognition	0.005 *	0.017 *	−0.011 *	−0.010 *
	Ecological knowledge	0.021 ***	0.078 ***	−0.052 ***	−0.048 ***
	Sample size	436			

***, **, and * indicate the 1%, 5%, and 10% significance levels, respectively.

## Data Availability

The data presented in this study are available on request from the corresponding author. The data are not publicly available due to personal privacy and non-open access to the research program.

## References

[1] Wang J., Wei X., Guo Q. (2018). A three-dimensional evaluation model for regional carrying capacity of ecological environment to social economic development: Model development and a case study in China. Ecol. Indic..

[2] Cai J., Li X., Liu L., Chen Y., Wang X., Lu S. (2021). Coupling and coordinated development of new urbanization and agro-ecological environment in China. Sci. Total Environ..

[3] Zhang K., Wen Z. (2008). Review and challenges of policies of environmental protection and sustainable development in China. J. Environ. Manag..

[4] Li Q., Ma H., Xu Z., Feng H., Bellingrath-Kimura S.D. (2022). Balancing socioeconomic development with ecological conservation towards rural sustainability: A case study in semiarid rural China. Int. J. Sustain. Dev. World Ecol..

[5] Qin S. (2011). Information technology strategy implementation based on differentiated competition on manufacturing enterprises in China. Proceedings of the 2011 2nd International Conference on Artificial Intelligence, Management Science and Electronic Commerce (AIMSEC).

[6] Guo H., Xu S., Pan C. (2020). Measurement of the spatial complexity and its influencing factors of agricultural green development in China. Sustainability.

[7] Chi Y., Zhou W., Wang Z., Hu Y., Han X. (2021). The influence paths of agricultural mechanization on green agricultural development. Sustainability.

[8] Möhring N., Wuepper D., Musa T., Finger R. (2020). Why farmers deviate from recommended pesticide timing: The role of uncertainty and information. Pest Manag. Sci..

[9] Guo H., Sun F., Pan C., Yang B., Li Y. (2021). The deviation of the behaviors of rice farmers from their stated willingness to apply biopesticides—A study carried out in Jilin Province of China. Int. J. Environ. Res. Public Health.

[10] Bagde S., Epple D., Taylor L. (2016). Does affirmative action work? Caste, gender, college quality, and academic success in India. Am. Econ. Rev..

[11] Pittock J., Bjornlund H., van Rooyen A. (2020). Transforming failing smallholder irrigation schemes in Africa: A theory of change. Int. J. Water Resour. Dev..

[12] Adegbeye M.J., Reddy P.R.K., Obaisi A.I., Elghandour M.M., Oyebamiji K.J., Salem A.Z. (2020). Sustainable agriculture options for production, greenhouse gasses and pollution alleviation, and nutrient recycling in emerging and transitional nations-An overview. J. Clean. Prod..

[13] Li C., Shi Y., Khan S.U., Zhao M. (2021). Research on the impact of agricultural green production on farmers’ technical efficiency: Evidence from China. Environ. Sci. Pollut. Res..

[14] Stern N., Stern N.H. (2007). The Economics of Climate Change: The Stern Review.

[15] Carof M., Colomb B., Aveline A. (2013). A guide for choosing the most appropriate method for multi-criteria assessment of agricultural systems according to decision-makers’ expectations. Agric. Syst..

[16] Thomas J. (2014). Rewarding bad behavior: How governments respond to terrorism in civil war. Am. J. Political Sci..

[17] Liu M., Zhang X. (2010). Farmers’ willingness on organic fertilizer application based on Logit model and influencing factors—A case of Shandong Province. Agric. Sci. Technol.-Hunan.

[18] Qiu X., Jin J., He R., Mao J. (2022). The deviation between the willingness and behavior of farmers to adopt electricity-saving tricycles and its influencing factors in Dazu District of China. Energy Policy.

[19] Li B., Ding J., Wang J., Zhang B., Zhang L. (2021). Key factors affecting the adoption willingness, behavior, and willingness-behavior consistency of farmers regarding photovoltaic agriculture in China. Energy Policy.

[20] Qu M., Zhao K., Zhang R., Gao Y., Wang J. (2022). Divergence between Willingness and Behavior of Farmers to Purchase Socialized Agricultural Services: From a Heterogeneity Perspective of Land Scale. Land.

[21] Han Y., Lyu H., Cheng S., He Y. (2022). Influencing mechanism and difference of poultry farmers’ willingness and behavior in breeding scale—Evidence from Jianghan Plain, China. Int. J. Environ. Res. Public Health.

[22] Fang X., Wang L., Sun C., Zheng X., Wei J. (2021). Gap between words and actions: Empirical study on consistency of residents supporting renewable energy development in China. Energy Policy.

[23] Bakker L., Sok J., Van Der Werf W., Bianchi F.J.J.A. (2021). Kicking the Habit: What makes and breaks farmers’ intentions to reduce pesticide use?. Ecol. Econ..

[24] Damalas C.A. (2021). Farmers’ intention to reduce pesticide use: The role of perceived risk of loss in the model of the planned behavior theory. Environ. Sci. Pollut. Res..

[25] Schultz T.W. (1966). Transforming traditional agriculture: Reply. J. Farm Econ..

[26] Li G., Fang C., Qiu D., Wang L. (2014). Impact of farmer households’ livelihood assets on their options of economic compensation patterns for cultivated land protection. J. Geogr. Sci..

[27] Wang J., Tao J., Yang C., Chu M., Lam H. (2017). A general framework incorporating knowledge, risk perception and practices to eliminate pesticide residues in food: A structural equation modelling analysis based on survey data of 986 Chinese farmers. Food Control.

[28] Gebre G.G., Isoda H., Rahut D.B., Amekawa Y., Nomura H. (2019). Gender differences in the adoption of agricultural technology: The case of improved maize varieties in southern Ethiopia. Women’s Stud. Int. Forum.

[29] Blan Y., Breiger R., Galaskiewicz J., Deborah D. (2005). Occupation, class, and social networks in urban China. Soc. Forces.

[30] Wang Y., Wang Y., Huo X., Zhu Y. (2015). Why some restricted pesticides are still chosen by some farmers in China? Empirical evidence from a survey of vegetable and apple growers. Food Control.

[31] Asafu-Adjaye J. (2006). Willingness to Adopt Soil Conservation Measures: A Case Study of Fijian Cane Farmers.

[32] Ervin D.E. (1982). Soil erosion control on owner-operated and rented cropland. J. Soil Water Conserv..

[33] Ajewole O.C. (2010). Farmer’s response to adoption of commercially available organic fertilizers in Oyo state, Nigeria. Afr. J. Agric. Res..

[34] Okello J.J., Okello R.M. (2010). Do EU pesticide standards promote environmentally-friendly production of fresh export vegetables in developing countries? The evidence from Kenyan green bean industry. Environ. Dev. Sustain..

[35] Zhang Y., Long H., Li Y., Ge D., Tu S. (2020). How does off-farm work affect chemical fertilizer application? Evidence from China’s mountainous and plain areas. Land Use Policy.

[36] Paudel K.P., Lohr L., Martin N.R. (2000). Effect of risk perspective on fertilizer choice by sharecroppers. Agric. Syst..

[37] Simtowe F. (2006). Can risk-aversion towards fertilizer explain part of the non-adoption puzzle for hybrid maize? Empirical evidence from Malawi. J. Appl. Sci..

[38] Wilson C., Dowlatabadi H. (2007). Models of decision making and residential energy use. Annu. Rev. Environ. Resour..

[39] Noll D., Dawes C., Rai V. (2014). Solar community organizations and active peer effects in the adoption of residential PV. Energy Policy.

[40] Chomać-Pierzecka E., Sobczak A., Urbańczyk E. (2022). RES Market Development and Public Awareness of the Economic and Environmental Dimension of the Energy Transformation in Poland and Lithuania. Energies.

[41] Waithaka M.M., Thornton P.K., Shepherd K.D., Ndiwa N.N. (2007). Factors affecting the use of fertilizers and manure by smallholders: The case of Vihiga, western Kenya. Nutr. Cycl. Agroecosyst..

[42] Qiao D., Li N.J., Cao L., Zhang D.S., Zheng Y., Xu T. (2022). How Agricultural Extension Services Improve Farmers’ Organic Fertilizer Use in China? The Perspective of Neighborhood Effect and Ecological Cognition. Sustainability.

[43] Li B., Shen Y. (2021). Effects of land transfer quality on the application of organic fertilizer by large-scale farmers in China. Land Use Policy.

[44] Kaliba A.R.M., Verkuijl H., Mwangi W. (2000). Factors affecting adoption of improved maize seeds and use of inorganic fertilizer for maize production in the intermediate and lowland zones of Tanzania. J. Agric. Appl. Econ..

[45] Nastis S.A., Mattas K., Baourakis G. (2019). Understanding farmers’ behavior towards sustainable practices and their perceptions of risk. Sustainability.

[46] Zhou H., Nanseki T., Song M., Chen T.G., Li D.P. (2014). Analysis on Factors Influencing Organic Fertilizer Use in China: A case study on wheat farmers in six eastern provincial–level regions. J. Fac. Agric. Kyushu Univ..

[47] Lu H., Hu L., Zheng W., Yao S., Qian L. (2020). Impact of household land endowment and environmental cognition on the willingness to implement straw incorporation in China. J. Clean. Prod..

[48] Zhao W.X., Xu Y.K. (2022). Public Expenditure and Green Total Factor Productivity: Evidence from Chinese Prefecture-Level Cities. Int. J. Environ. Res. Public Health.

[49] Li Y.C., Fan Z.Y., Jiang G.H., Quan Z. (2021). Addressing the Differences in Farmers’ Willingness and Behavior Regarding Developing Green Agriculture—A Case Study in Xichuan County, China. Land.

[50] Hwang Y., Kim D.J. (2007). Understanding affective commitment, collectivist culture, and social influence in relation to knowledge sharing in technology mediated learning. IEEE Trans. Prof. Commun..

[51] Conley T.G., Udry C.R. (2010). Learning about a new technology: Pineapple in Ghana. Am. Econ. Rev..

[52] Aida T. (2017). Neighbourhood effects in pesticide use: Evidence from the rural Philippines. J. Agric. Econ..

[53] Feder G., Slade R. (1986). A comparative analysis of some aspects of the training and visit system of agricultural extension in India. J. Dev. Stud..

[54] Abhilash P.C., Singh N. (2009). Pesticide use and application: An Indian scenario. J. Hazard. Mater..

[55] Antczak E. (2021). Analyzing Spatiotemporal Development of Organic Farming in Poland. Sustainability.

[56] Fan L.X., Niu H.P., Yang X.M., Qin W., Bento C.P.M., Ritsema C.J., Geissen V. (2015). Factors affecting farmers’ behaviour in pesticide use: Insights from a field study in northern China. Sci. Total Environ..

[57] Yu L.Y., Liu H.D., Diabate A., Qian Y.Y., Sibiri H., Yan B. (2020). Assessing influence mechanism of green utilization of agricultural wastes in five provinces of china through farmers’ motivation-cognition-behavior. Int. J. Environ. Res. Public Health.

[58] Zhang Y.X., Halder P., Zhang X.N., Qu M. (2020). Analyzing the deviation between farmers’ Land transfer intention and behavior in China’s impoverished mountainous Area: A Logistic-ISM model approach. Land Use Policy.

[59] Zhou B.Y., Liu W.X., Lu W.N., Zhao M.J., Li L.F. (2020). Application of OECD LSE framework to assess spatial differences in rural green development in the Arid Shaanxi Province, China. Int. J. Environ. Res. Public Health.

[60] Xu T., Ni Q., Yao L.Y., Qiao D., Zhao M.J. (2020). Public preference analysis and social benefits evaluation of river basin ecological restoration: Application of the choice experiments for the shiyang river, China. Discret. Dyn. Nat. Soc..

[61] Qiao D., Li W.Q., Zhang D.S., Yan Y., Xu T. (2022). How do You Want to restore?—Assessing the Public Preferences and Social Benefits of Ecological Restoration for Natural Rubber Plantation in China. Front. Environ. Sci..

[62] Liu Y.T., Chen M.Q., Xie X.X. (2021). Study on Deviation between Farmers’ Willingness to Adopt Ecological Farming and Their Behaviors of Jiangxi Province. Areal Res. Dev..

